# Impact of Clinical Characteristics and Treatment on Cholangiocarcinoma Prognosis in Southern Thailand

**DOI:** 10.1002/cam4.70491

**Published:** 2024-12-18

**Authors:** Chirawadee Sathitruangsak, Tanawat Pattarapuntakul, Apichat Kaewdech, Tortrakoon Thongkan, Apinya Prisutkul, Phatcharaporn Thongwatchara, Hutcha Sriplung, Chanon Kongkamol, Kanyanatt Kanokwiroon, Sumalee Obchoei, Pimsiri Sripongpun

**Affiliations:** ^1^ Holistic Center for Cancer Study and Care (HOCC‐PSU) and Medical Oncology Unit, Division of Internal Medicine, Faculty of Medicine Prince of Songkla University Hat Yai, Songkhla Thailand; ^2^ Gastroenterology and Hepatology Unit, Division of Internal Medicine, Faculty of Medicine Prince of Songkla University Hat Yai, Songkhla Thailand; ^3^ Department of Surgery, Faculty of Medicine Prince of Songkla University Hat Yai, Songkhla Thailand; ^4^ Epidemiology Unit, Faculty of Medicine Prince of Songkla University Hat Yai, Songkhla Thailand; ^5^ Division of Digital Innovation and Data Analytics, Faculty of Medicine Prince of Songkla University Songkhla Thailand; ^6^ Department of Biomedical Sciences and Biomedical Engineering, Faculty of Medicine Prince of Songkla University Hat Yai, Songkhla Thailand; ^7^ Division of Health and Applied Sciences, Biochemistry Graduate Program, Faculty of Science Prince of Songkla University Hat Yai, Songkhla Thailand

**Keywords:** bile duct cancer, hilar, intrahepatic, periductal, prognosis, survival

## Abstract

**Background:**

Cholangiocarcinoma (CCA) is most commonly seen in Northeastern Thailand and other parts of Asia where liver flukes are prevalent. However, it is unknown whether CCA patients in low and high liver fluke prevalence areas are similar. This study aimed to analyze the clinical characteristics and outcomes of CCA patients in Southern Thailand.

**Methods:**

We retrospectively reviewed 223 patients diagnosed with CCA between 2018 and 2021 in a tertiary‐care center. Clinicopathologic data were reviewed and compared between intrahepatic, perihilar, and distal CCA (iCCA, pCCA, and dCCA, respectively). Overall survivals (OS) were determined by Kaplan–Meier method and multivariable Cox regressions.

**Results:**

The mean age was 63.9 years; 50.7% were men. The most common subtype was iCCA (49.3%), followed by pCCA (36.3%) and dCCA (14.3%). Most patients were diagnosed at a later stage: 59.4% TMN stage IV and 23.3% stage III. Cirrhosis was present in 6.3%, while the presence of liver fluke was not detected. Only 15.1% of the cohort were deemed resectable. The median OS for iCCA, pCCA, and dCCA patients were 27.3, 22.0, and 19.3 weeks, respectively (*p* = 0.9). One‐year survival rate differed significantly between resectable and unresectable patients (85.2% vs. 21.2%, *p* < 0.0001). TMN stage (aHR 1.88), palliative biliary drainage (aHR 0.31), and systemic chemotherapy (aHR 0.19) were independent predictors for mortality in unresectable pCCA and dCCA patients. In unresectable iCCA patients, only systemic chemotherapy was significant (aHR 0.30).

**Conclusion:**

Most patients were diagnosed late, and the median OS was only 5–6 months. Unresectable CCA patients with systemic chemotherapy and palliative biliary drainage had better survival rates.

## Graphical abstract

TMN stage, palliative biliary drainage, and systemic chemotherapy were predictors for mortality in unresectable cholangiocarcinoma.

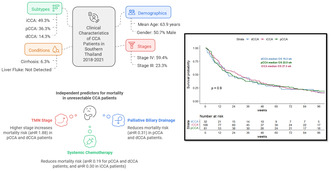

**Abbreviations**
AFP: alpha‐fetoprotein
AJCC: American Joint Committee on Cancer staging system
ALP: alkaline phosphatase
ALT: alanine aminotransferase
AST: aspartate transaminase
CA 19–9: carbohydrate antigen 19–9
CCA: cholangiocarcinoma
CEA: carcinoembryonic antigen
dCCA: distal cholangiocarcinoma
eCCA: extrahepatic cholangiocarcinoma
ERCP: endoscopic retrograde cholangiopancreatography
HBV: hepatitis B virus
HCC: hepatocellular carcinoma
HCV: hepatitis C virus
IBD: inflammatory bowel disease
iCCA: intrahepatic cholangiocarcinoma
INR: International Normalized Ratio
OS: overall survival
OV: *Opisthorchis viverrini*
pCCA: perihilar cholangiocarcinoma
PSC: primary sclerosing cholangitis
PTBD: percutaneous transhepatic biliary drainage

## 1 Introduction

Cholangiocarcinoma (CCA) is a heterogeneous group of malignant tumors arising from the biliary tracts, excluding the gallbladders and ampulla of Vater. CCA are commonly classified into two main groups based on their anatomical site of origin; intrahepatic CCA (iCCA) and extrahepatic CCA (eCCA). And eCCA can further be subdivided into perihilar CCA (pCCA), and distal CCA (dCCA) [1, 2].

CCA is an uncommon cancer in Western countries, estimates of occurrence vary between 0.3 and 6 cases per 100,000 annually [1]. On the contrary, Thailand has the highest incidence of CCA in the world, accounted for 22.9 cases per 100,000 persons per year [3]. However, the incidences of CCA are varying in different areas of Thailand. The Northeast region had the highest number of CCA patients (61.5%), followed by the North (17%), and Central regions (13.4%) [4], whereas Bangkok and the South had a relatively low number of CCA patients (2.8% and 2.7%, respectively) [4]. The annual incidence rate was also significantly varied. In 2013, the incidence of CCA was 28.83 and 2.98 cases per 100,000 persons in the Northeast and the South, respectively [4].

The majority of CCA patients occur sporadically, but there are several well‐known risk factors. Chronic biliary tract infection is prevalent in Southeast Asia. Previous studies showed the frequency of CCA cases in each region of Thailand was correlated with the reported prevalence of *Opisthorchis viverrini* (OV) infection (up to 67% in the Northeast and only 0.1% in the South) [5, 6]. A prospective case‐controlled study conducted in northeastern Thailand also demonstrated that patients with OV infection had a higher incidence of CCA than non‐infected patients [7]. These discrepancies reflect distinct epidemiological characteristics among the populations in each region, and possibly differences in the treatment outcomes.

Given the heterogeneity within CCA and their difference in etiology, the treatment outcome was various. Nevertheless, these different types of CCAs have been treated as a single disease, with rather dissatisfying outcomes. The only potentially curative treatment for CCA is surgical resection; however, only 10%–40% of CCA are resectable at the time of diagnosis [8, 9]. Although progress has been made in the treatment of metastatic CCA, the survival rate is still limited. The 5‐year survival is only 7%–20% and the recurrence rate after curative‐intent surgery is still disappointing [1, 10].

The purpose of this study was to better determine the clinical characteristics of CCA in Southern Thailand and to evaluate the treatment outcomes on CCA patients in the real‐world setting where OV was not prevalent.

## 2 Materials and Methods

### 2.1 Patients and Data Collection

This is a retrospective study conducted at Songklanagarind hospital, a tertiary care hospital and the only university hospital in Southern part of Thailand. We searched all adult patients with International Classification Diagnosis 10th edition (ICD‐10) diagnostic codes for malignant neoplasm of liver and intrahepatic bile ducts (codes C22.0–C22.9) and malignant neoplasm of other and unspecified parts of biliary tract (codes C24.0–C24.9) from January 2018 through June 2021. The diagnoses were indexed and retrieved from the Hospital Information System (HIS) for the study. The pathological diagnosis and radiographic reports were individually reviewed to confirm the diagnosis of CCA and only patients with the final diagnosis of CCA were included. We classified all tumors on the basis of histology and/or cytology results for patients who underwent tissue biopsy. For patients in whom tumor biopsy was not performed, we used information on clinical, laboratory and radiological characteristics to determine the final diagnosis. The clinical characteristics, laboratory results, imaging data, endoscopic interventions, treatment details and outcomes were retrieved for analysis.

Tumor stage was categorized according to the American Joint Committee on Cancer (AJCC) staging system 8th edition. Treatment failure was defined as radiographic progression either local failure or distant metastasis. Date of death was obtained from the death certificate using data from the Thailand Civil Registration Database. Patients were excluded if they had no follow‐up information.

### 2.2 Statistical Analysis

The overall survival (OS) was defined as the duration from the date of diagnosis to death. The probabilities of OS were estimated using the Kaplan–Meier and multivariable Cox regressions. The difference in OS among CCA subgroups was assessed using the log‐rank test.

A descriptive analysis was calculated using all available data. Aggregated data were presented as frequency (percentage), mean ± standard deviation (SD) or median with interquartile range (IQR) as appropriate. The *t*‐test or Wilcoxon rank sum test was used to determine differences for continuous variable, and the two‐sided Fisher's exact or chi‐squared test were used in the comparison of categorical variables. Univariate analysis of each independent variable was performed using simple logistic regression. Statistical analyses were performed using R program version 4.1.1. A *p* value of < 0.05 was considered statistically significant.

## 3 Results

**FIGURE 1:**
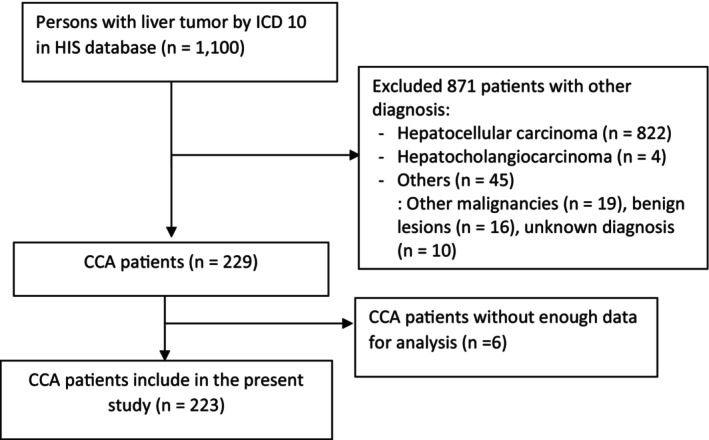
CONSORT diagram of the study.

During the 3.5‐year study period, a total of 1100 patients diagnosed with malignant neoplasm of liver and bile ducts were evaluated at Songklanagarind hospital, Songkhla, Thailand. Of those, 874 cases were excluded: 822 hepatocellular carcinomas, 19 other malignancies, 16 benign lesions, 10 unknown diagnoses, 4 hepatocholangiocarcinoma, and 6 with inadequate data on medical records (shown in Figure 1).

### 3.1 Baseline Characteristics

A total of 223 CCA patients were enrolled, including 110 patients (49.3%) with iCCA, 81 patients (36.3%) with pCCA, and 32 patients (14.3%) with dCCA. The clinical characteristics were summarized in Table 1. Of these 223 patients, 113 (50.7%) were men, and the median age at diagnosis was 63.9 ± 11.9 years. Histopathologically, adenocarcinoma subtype represented for the majority of CCA (80.4%). Prior to any treatment modality, pCCA patients had the highest incidence of cholangitis (59.2%), followed by dCCA patients (34.4%), and iCCA patients (25.2%) (*p* < 0.001). There were no differences in smoking and alcohol consumption between subgroups. High percentage of non‐smokers (57.2%) and non‐alcoholic drinkers (63.9%) in our CCA cohort were reported. The common co‐morbidities were hypertension (36.3%), hyperlipidemia (22.9%), and type 2 diabetes mellitus (17%).

**TABLE 1:** Demographic characteristics of CCA patients.

	Total (*n* = 223)	iCCA (*n* = 110)	pCCA (*n* = 81)	dCCA (*n* = 32)	*p*
Gender (male:female)	113:110	50:60	45:36	18:14	0.306
Age at diagnosis (mean ± SD)	63.9 ± 11.9	62.3 ± 11.9	65 ± 12.1	67 ± 10.4	0.87
Body Mass Index, BMI (median [IQR])	22 (19, 24.6)	21.9 (19, 24.6)	21.6 (19.2, 25)	22 (19.4, 23)	0.909
Smoking status (*n* (%))					0.805
Current smoker	53 (26.4)	25 (25.5)	20 (26.7)	8 (28.6)	
Ex‐smoker	33 (16.4)	15 (15.3)	15 (20)	3 (10.7)	
Never smoker	115 (57.2)	58 (59.2)	40 (53.3)	17 (60.7)	
Alcohol consumption (*n*, %)					0.391
Current drinker	34 (16.8)	20 (20.2)	11 (14.7)	3 (10.7)	
Ex‐drinker	31 (15.3)	10 (10.1)	15 (20)	6 (21.4)	
Social drinker	8 (4)	5 (5.1)	3 (4)	0 (0)	
Never	129 (63.9)	64 (64.6)	46 (61.3)	19 (67.9)	
Family history of malignancy (*n*, %)	40 (18.4)	22 (20.6)	12 (15)	6 (20)	0.672
Underlying disease (*n*, %)					
Cirrhosis	14 (6.3)	10 (9.1)	2 (2.5)	2 (6.2)	0.176
Choledochal cyst	3 (1.3)	1 (0.9)	1 (1.2)	1 (3.1)	0.538
Cholelithiasis	1 (0.4)	0 (0)	0 (0)	1 (3.1)	0.143
Hyperlipidemia	51 (22.9)	21 (19.1)	22 (27.2)	8 (25)	0.403
Type 2 diabetes mellitus	38 (17)	19 (17.3)	11 (13.6)	8 (25)	0.346
Hypertension	81 (36.3)	39 (35.5)	33 (40.7)	9 (28.1)	0.438
History of cholangitis (*n*, %)	82 (38.9)	26 (25.2)	45 (59.2)	11 (34.4)	< 0.001
Venous thromboembolism (*n*, %–available in 200 patients)	38 (19)	24 (23.5)	11 (15.3)	3 (11.5)	0.229

For the underlying diseases that might predispose to CCA development, 14 patients (6.3%) had histological or clinical evidence of liver cirrhosis; iCCA patients had a higher rate of cirrhosis (9.1%), compared with dCCA (6.2%) and pCCA (2.5%), but not statistically significant (*p* = 0.176). Overall, the co‐morbidities were not significantly different between subgroups. In an entire CCA cohort, three patients (1.3%) had a known history of choledochal cyst and only 1 patient (0.4%) had cholelithiasis, but there were no patients with history of primary sclerosing cholangitis (PSC) or inflammatory bowel disease (IBD) in our cohort. In addition, there were no patients with known or suspected history of biliary parasitic disease (e.g., OV infection). Forty patients (18.4%) had a history of malignancy in their family members.

In our CCA cohort, the overall rate of venous thromboembolism at the time of diagnosis was 19% (1.0% pulmonary embolism, 2.5% deep vein thrombosis, and 15.0% visceral or splanchnic thrombosis), which was the highest in iCCA (23.5%), compared with pCCA (15.3%) and dCCA (11.5%) but not statistically significant (*p* = 0.229).

### 3.2 Presenting Signs and Symptoms

At the time of diagnosis, all pCCA and dCCA patients had abnormal symptoms. Only 5 of the 110 iCCA patients (4.5%) were asymptomatic at diagnosis, which was found incidentally or during investigation of abnormal liver enzymes. Presenting symptoms, in decreasing order, included weight loss (72.9%), abdominal pain (61%), jaundice (57.8%), anorexia (48.9%), pruritus (26.5%), fever (14.3%), nausea and/or vomiting (10.3%), and palpable mass (4.9%) (Table 2).

**TABLE 2:** Clinical presentation of CCA patients.

	Total (*n* = 223)	iCCA (*n* = 110)	pCCA (*n* = 81)	dCCA (*n* = 32)	*p*
Asymptomatic (incidental finding)	5 (2.2)	5 (4.5)	0	0	0.102
Jaundice	129 (57.8)	28 (25.5)	74 (91.4)	27 (84.4)	< 0.001
Abdominal pain	136 (61)	83 (75.5)	34 (42)	19 (59.4)	< 0.001
Anorexia	109 (48.9)	49 (44.5)	45 (55.6)	15 (46.9)	0.313
Weight loss					0.543
< 5%	17 (7.7)	9 (8.3)	5 (6.2)	3 (9.7)	
More than 5%	144 (65.2)	68 (62.4)	52 (64.2)	24 (77.4)	
No weight loss	25 (11.3)	15 (13.8)	8 (9.9)	2 (6.5)	
Unknown	35 (15.8)	17 (15.6)	16 (19.8)	2 (6.5)	
Pruritus	59 (26.5)	8 (7.3)	39 (48.1)	12 (37.5)	< 0.001
Fever	32 (14.3)	16 (14.5)	11 (13.6)	5 (15.6)	0.958
Palpable mass	11 (4.9)	11 (10)	0	0	0.002
Nausea/Vomiting	23 (10.3)	13 (11.8)	8 (9.9)	2 (6.2)	0.651

The patients with eCCA had the highest rate of jaundice (91.4% in pCCA and 84.4% in dCCA) compared with iCCA patients (25.5%) (*p* < 0.001). Correspondingly, the pruritus was reported mostly in eCCA patients (48.1% in pCCA and 37.5% in dCCA) compared with iCCA patients (7.3%) (*p* < 0.001).

On the other hand, the iCCA groups had a significant higher rate of abdominal pain (75.5%) compared with dCCA (59.4%) and pCCA (42%) (*p* < 0.001). Ten percent of iCCA patients presented with palpable mass, while none of dCCA and pCCA patients had palpable mass at the time of diagnosis.

About half of CCA patients had anorexia (48.9%), and around two‐third (65.2%) experienced weight loss more than 5% at the time of diagnosis. There were no differences in the rate of fever, anorexia, nausea and vomiting between subgroups.

In the entire cohort, patients had a high proportion of stage III (23.3%) and stage IV (59.4%) disease at the time of diagnosis; iCCA patients had the highest proportion of metastatic disease (70.9%), followed by dCCA (59.4%), and pCCA (39.5%; *p* < 0.001) (Table 3). Median tumor size was also significantly different between subtypes; 8.4, 3.6, and 3.2 cm for iCCA, pCCA, and dCCA, respectively (*p* < 0.001). The most common metastatic site in overall CCA cohort was liver (35.4%), followed by intraabominal lymph nodes (29.6%), and lung (20.2%).

**TABLE 3:** Staging at diagnosis.

	Total (*n* = 223)	iCCA (*n* = 110)	pCCA (*n* = 81)	dCCA (*n* = 32)	*p*
Staging (AJCC 8th edition) [data available for complete staging in 219 patients]					< 0.001
Stage I	13 (5.9)	11 (10)	2 (2.5)	0	
Stage II	25 (11.4)	10 (9.1)	9 (11.2)	6 (20.7)	
Stage III	51 (23.3)	12 (10.9)	35 (43.8)	4 (13.8)	
Stage IV	130 (59.4)	77 (70)	34 (42.5)	19 (65.5)	
Size of primary tumor (median [IQR], cm)	5.1 (3.3, 9)	8.4 (5.2, 10.4)	3.6 (2.7, 4.9)	3.2 (2.2, 4.8)	< 0.001
Presence of metastatic disease	129 (57.8)	78 (70.9)	32 (39.5)	19 (59.4)	< 0.001
Site of metastasis					
Lung	45 (20.2)	31 (28.2)	6 (7.4)	8 (25)	0.001
Liver	79 (35.4)	54 (49.1)	13 (16)	12 (37.5)	< 0.001
Abdominal LN	66 (29.6)	39 (35.5)	18 (22.2)	9 (28.1)	0.138
Bone	11 (4.9)	9 (8.2)	2 (2.5)	0	0.11
Others	51 (22.9)	25 (22.7)	19 (23.5)	7 (21.9)	0.983

Abbreviation: AJCC, American Joint Committee on Cancer staging system.

In comparison with iCCA patients, patients with eCCA had a significantly higher rate of abnormal liver biochemistries, including jaundice, elevated aspartate transaminase (AST) and alanine aminotransferase (ALT), and elevated alkaline phosphatase (ALP) (*p* < 0.001). The serum albumin and globulin level were also significantly lower in eCCA (*p* = 0.019 and 0.009, respectively). Other than that, the International Normalized Ratio (INR) was also significantly higher in eCCA patients compared with iCCA (*p* < 0.001). However, iCCA patients had significantly lower platelet counts, compared with pCCA and dCCA patients (*p* < 0.001). There were no significant differences in baseline hemoglobin, white blood cell counts, and serum creatinine at the time of diagnosis (Table 4).

**TABLE 4:** Baseline laboratory results at the time of diagnosis.

	Total (*n* = 223)	iCCA (*n* = 110)	pCCA (*n* = 81)	dCCA (*n* = 32)	*p*
Serum tumor marker (median, IQR)					
CA 19–9 (U/mL)	356 (30.9, 2213.5)	54.5 (14.8, 1390.2)	602.3 (135, 2271.5)	914.5 (313.4, 2761.5)	0.006
CEA (ng/mL)	4.4 (2.2, 26.8)	4.3 (2.1, 44.3)	4.2 (2.4, 10.6)	5.2 (2.9, 10.6)	0.689
AFP (ng/mL)	3.2 (2.2, 7)	3.2 (2.3, 7.4)	3.4 (2.4, 4.1)	1.9 (1.6, 3.8)	0.22
Total bilirubin (mg/dL) (median, IQR)	10.4 (0.8, 21.4)	0.8 (0.4, 8.2)	19.6 (12, 25.3)	18.4 (8, 22.1)	< 0.001
AST (U/L) (median, IQR)	73 (45.8, 124.5)	55 (32, 101)	93 (60.2, 151.2)	112 (64, 175)	< 0.001
ALT (U/L) (median, IQR)	49.5 (27, 86)	32 (19, 65)	56.5 (36.5, 97.2)	73 (48, 128.5)	< 0.001
ALP (U/L) (median, IQR)	362 (211.8, 587.8)	276 (144, 469)	402 (287.8, 638)	461 (315.5, 736)	< 0.001
Albumin (g/dL) (median, IQR)	3.4 (2.9, 3.9)	3.6 (2.9, 4.1)	3.3 (2.8, 3.7)	3.2 (2.9, 3.7)	0.019
Globulin (g/dL) (median, IQR)	3.7 (3.3, 4.2)	3.8 (3.5, 4.2)	3.4 (3.2, 4.1)	3.6 (3.2, 4)	0.009
Hemoglobin (g/dL) (median, IQR)	11 (9.9, 12.2)	11.4 (9.9, 12.5)	10.8 (9.9, 11.7)	11.2 (9.9, 12.3)	0.204
White blood cell count (μL) (median, IQR)	10200 (7900, 12800)	10710 (8035, 13080)	9390 (7882.5, 11725)	10250 (8237.5, 12412.5)	0.247
%PMN (median, IQR)	76.2 (68, 82)	77 (69.4, 83.3)	74.7 (66.5, 80.5)	77.7 (68.1, 83.5)	0.369
Platelet count (×10^3^/μL) (median, IQR)	309 (236, 385)	265 (202.5, 341)	342 (276.8, 434.8)	319 (280.8, 376.2)	< 0.001
INR (s) (median, IQR)	1.2 (1.1, 1.5)	1.2 (1.1, 1.3)	1.4 (1.2, 1.8)	1.3 (1.1, 1.6)	< 0.001
Creatinine (mg/dL) (median, IQR)	0.8 (0.6, 1)	0.7 (0.6, 0.9)	0.8 (0.6, 1)	0.8 (0.6, 1)	0.692
Serology					
HBsAg+ (available in 196 patients)	9 (9.4)	6 (11.1)	3 (9.1)	0	0.88
Anti HBc+ (available in 110 patients)	48 (44.4)	28 (43.1)	18 (50)	2 (28.6)	0.542
Anti HCV+ (available in 180 patients)	6 (3.4)	3 (3.2)	3 (5.2)	0	0.614
Anti HIV+ (available in 164 patients)	3 (1.8)	1 (1.2)	1 (1.7)	1 (4.5)	0.525

Abbreviations: AFP, alpha‐fetoprotein; ALP, alkaline phosphatase; ALT, alanine aminotransferase; AST, aspartate transaminase; CA 19–9, carbohydrate antigen 19–9; CEA, carcinoembryonic antigen; INR, International Normalized Ratio.

Regarding serum tumor markers, elevated levels of serum carbohydrate antigen 19–9 (CA19‐9) were more frequently observed in dCCA and pCCA than iCCA subgroup. Among 187 patients with available CA19‐9 levels, the positive rates for CA19‐9 (beyond upper limit of normal level at our center) were 88.5% in dCCA, 83.6% in pCCA, and 59.8% in iCCA patients. Serum CA19‐9 levels were highest in dCCA patients, with a median of 914.5 U/mL (IQR, 313.4–2761.5 U/mL). This was followed by pCCA patients (median, 602.3 U/mL; IQR, 135–2271.5 U/mL) and iCCA patients (median, 54.5 U/mL; IQR, 14.8–1390.2 U/mL; *p* = 0.006). However, there were no significant differences in serum carcinoembryonic antigen (CEA) and alpha‐fetoprotein (AFP) levels among subgroups (*p* = 0.689 and 0.22, respectively).

The iCCA cohort (*N* = 110) was further subclassified into perihilar (*N* = 51) and peripheral (*N* = 55) iCCA based on imaging characteristics [11]; four patients were unable to subclassified as there were no imagings to review for the subclassification. While no significant differences in baseline characteristics between these two subclassification were observed, patients with perihilar iCCA presented more frequently with cholangitis (35.3% vs. 14.6%, *p* = 0.032), which corresponded with higher rates of jaundice (37.3% vs. 14.8%, *p* = 0.016), fever (23.5% vs. 7.4%, *p* = 0.043), and pruritus (13.7% vs. 1.9%, *p* = 0.028). Furthermore, perihilar iCCA was associated with significantly elevated levels of total bilirubin, AST, ALT, and ALP (*p* = 0.007, 0.013, and 0.006, respectively) (Table S1). No significant differences were found between the two groups with respect to tumor stage (*p* = 0.085), and primary tumor size at diagnosis (*p* = 0.156).

Serologic evidence of chronic hepatitis B virus (HBV) infection was positive in 48 patients (44.4%). pCCA and iCCA patients had high rates of having chronic/past HBV infection (50% and 43.1%, respectively), compared with dCCA patients (28.6%), but without statistical significance (*p* = 0.542). Six patients (3.4%) had serologic evidence of chronic hepatitis C virus (HCV) infection.

### 3.3 Treatment and Outcomes

At the time of diagnosis, only 15.1% of the entire CCA cohort were deemed resectable. And none of the patients in our cohort received immunotherapy or liver transplantation. Of the 34 patients who underwent surgical resection, 9 patients (26.5%) had preoperative cholangitis; 15 patients (44.1% of the 34 patients) underwent preoperative biliary drainage, consisting of endoscopic retrograde cholangiopancreatography (ERCP) with stent placement in 9 patients (26.5%) and percutaneous transhepatic biliary drainage (PTBD) in 6 patients (17.6%). The mean total bilirubin level at diagnosis was 11.6 ± 11.2 mg/dL for those who underwent curative resection. Among the surgical cohort, 21 patients (61.8%) had eCCA, comprising 14 patients (41.2%) with pCCA and 7 patients (20.6%) with dCCA. Ten patients (29.4%) received adjuvant chemotherapy. None of the patients received neoadjuvant chemotherapy.

Within the cohort of patients with unresectable disease, 48 patients (22.5%) were treated with palliative chemotherapy. The most common first‐line chemotherapy regimen utilized was platinum‐based in combination with gemcitabine (54.2%), followed by platinum‐based therapy with fluoropyrimidine (33.3%). Monotherapy with fluoropyrimidine (4.2%) or gemcitabine (2.1%) was less frequently used.

**FIGURE 2:**
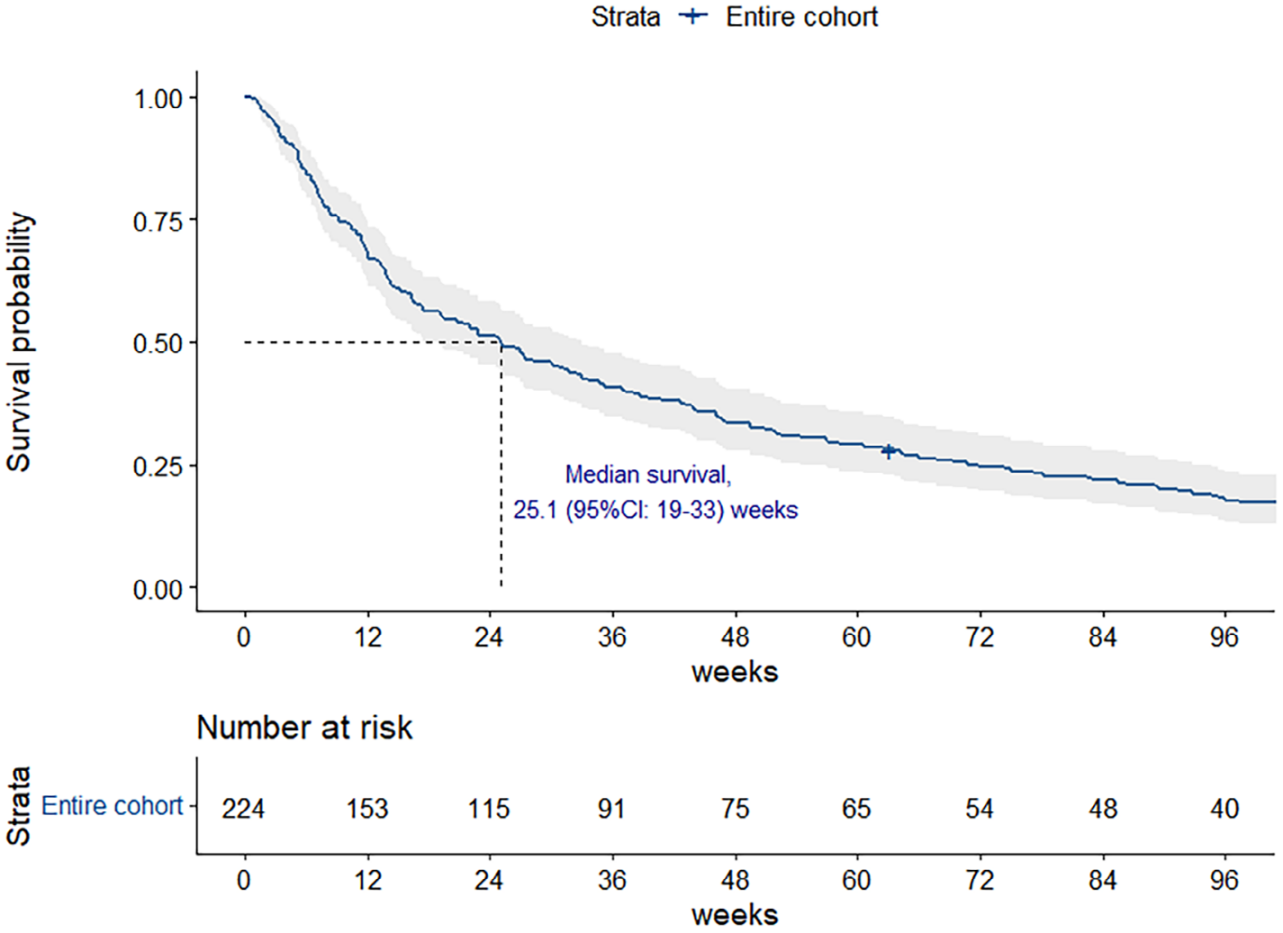
Kaplan–Meier survival curve for overall CCA patients. Kaplan–Meier estimates showed overall survival, with median values of 25.1 weeks in all CCA patients.

**FIGURE 3:**
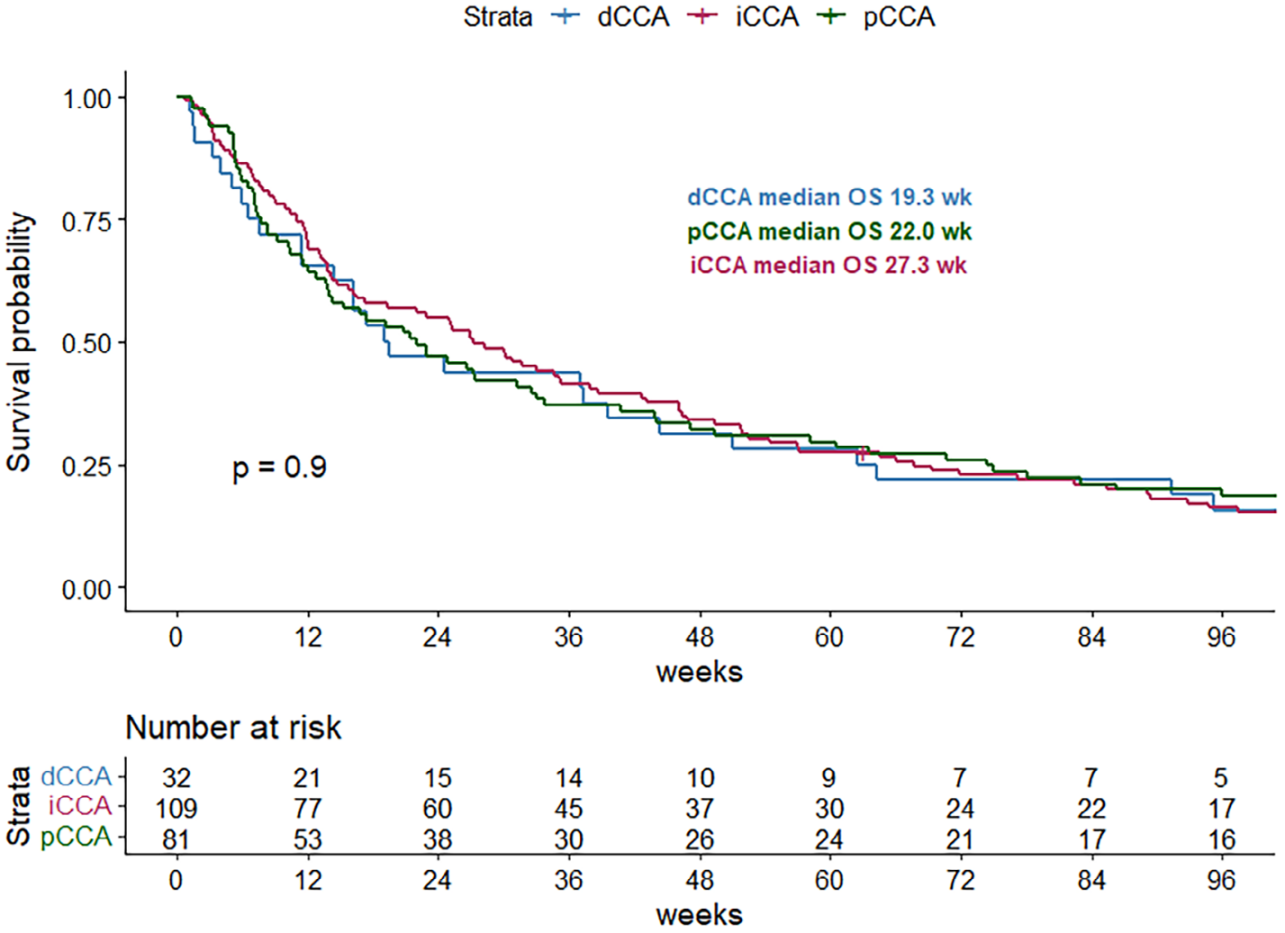
Kaplan–Meier survival curve for CCA patients according to location of primary tumors.

**FIGURE 4:**
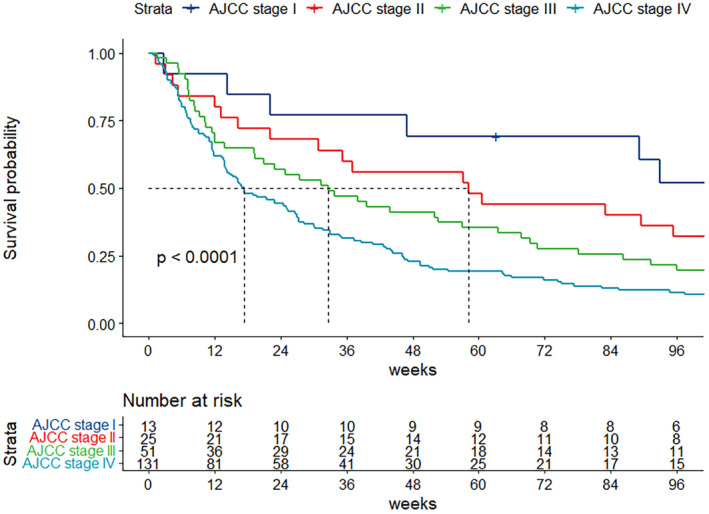
Kaplan–Meier survival curve for CCA patients according to AJCC stage in overall study population.

At the median follow‐up time of 51 months (95% CI: 49–54 months), the median survival time was 25.1 weeks (95% CI: 19–33 weeks) (shown in Figure 2). We performed exploratory analyses to identify the association between location of primary tumor and the OS of the patients with CCA. The patients of all stages (stage I–IV) were merged in analysis to identify the prognostic relevance of the location of tumor. The location of primary tumor was not correlated with prognosis. The median OS was 27.3, 22.0, and 19.3 weeks in iCCA, pCCA, and dCCA subgroups, respectively (*p* = 0.9) (shown in Figure 3). Within the iCCA cohort, no significant difference in overall survival was observed between patients with perihilar iCCA and peripheral iCCA (*p* = 0.46) (Figure S1). We further analyzed the correlation between tumor staging and the OS. Patients with higher stages had significantly inferior OS. The 1‐year survival rates were 69.2%, 56.0%, 39.2%, and 19.9% for stages I, II, III, and IV, respectively (shown in Figure 4).

**FIGURE 5:**
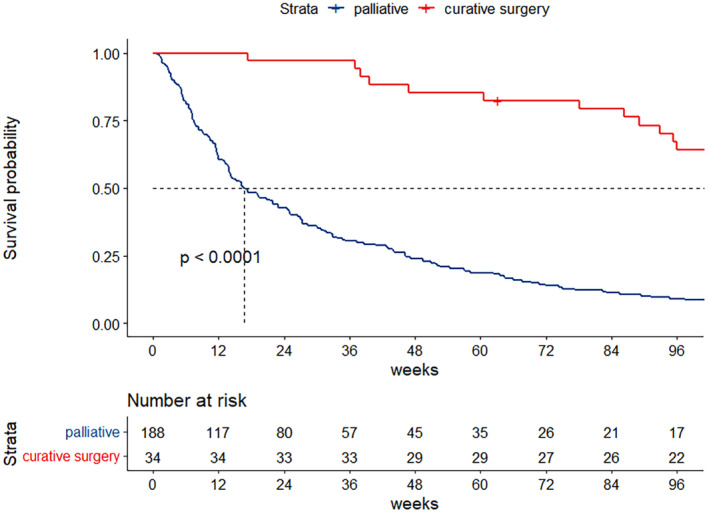
Kaplan–Meier survival curve for CCA patients according to curative surgery.

Regarding the surgical outcome, the curative resected CCA group had a significant better 1‐year survival rate, 85.2%, compared with non‐resected groups, 21.2% (*p* < 0.0001). The median OS for resected CCA in our cohort was not established, as more than half of the patients who underwent curative surgery were still living at the time of analysis (shown in Figure 5). However, the median OS for non‐resected CCA was only 16.7 weeks (95% CI: 13.9–24.6 weeks).

### 3.4 Factors Associate With Prognosis

In an adjusted multivariable Cox regressions analysis, surgical resection was associated with better outcome for CCA patients (HR 0.18; 95% CI, 0.11–0.29; *p* < 0.001). There were no significant differences in OS with respect to location of primary tumor, mean age (*p* = 0.5), or sex (*p* = 0.6). Among those who underwent curative resection, the OS was not different between patients with or without preoperative biliary drainage (*p* = 0.7 by log‐rank test).

**FIGURE 6:**
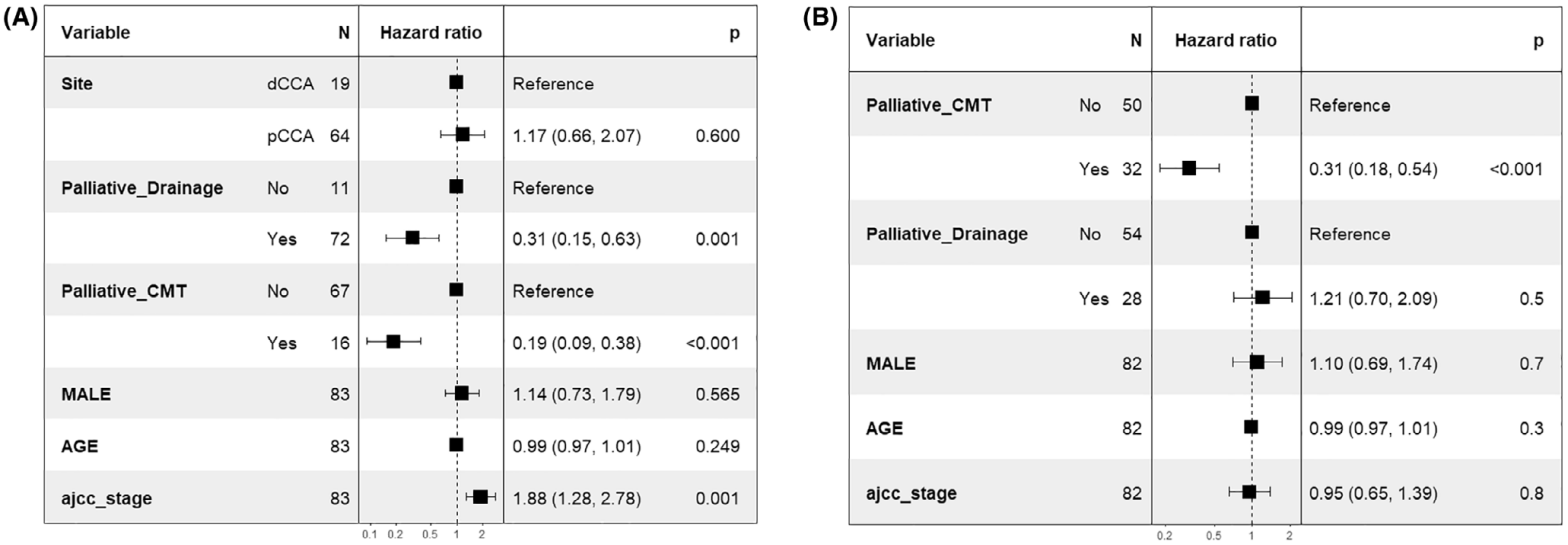
Factors associated with long‐term overall survival in eCCA (6A) and iCCA (6B).

As most of the patients in the cohort were in advanced, unresectable stage, we were interested in exploring the factors associated with the OS in such group of patients. Among patients with unresectable eCCA (dCCA and pCCA), after adjustment with age, sex, and AJCC staging, the OS was significantly improved with palliative biliary drainage (HR 0.31; 95% CI, 0.15–0.63; *p* = 0.001) and systemic chemotherapy (HR 0.19; 95% CI, 0.09–0.38; *p* < 0.001) (shown in Figure 6A).

Whereas in the iCCA subgroup, systemic chemotherapy was the only independent factor associated with a better survival outcome (HR 0.30; 95% CI, 0.18–0.52; *p* < 0.001) (shown in Figure 6B).

## 4 Discussion

In this study, we evaluated the clinical characteristics and prognostic relevance in CCA patients in Southern Thailand, representing a non‐endemic area for liver fluke infection. We collected data of 223 patients diagnosed with CCA within 3.5 years of time. Diagnosis was mainly confirmed by histopathology (77%), which was much higher than previous reported from the Northeastern Thailand (only 9% of all cases of liver diseases), despite being the highest reported CCA incidence globally [1]. No evidence of OV infection was observed within our cohort. This finding is consistent with previous epidemiological reports indicating a low prevalence (0%–0.01%) of OV infection in southern Thailand compared to that of other parts of the country (19.3% in the north, 15.7% in the northeast, 3.8% in the central region) [5].

The primary location of CCA varies among regions in the world. In United States, pCCA was a major type (50%–60%), followed by dCCA (20%–30%), and iCCA (10%–20%) [1]. On the contrary, iCCA was the largest group in our CCA cohort, accounting for 49.3% of all CCA, followed by pCCA (36.3%), and dCCA (14.3%). These distributions were also correlated with previous reported from German, Korea, and the Northeastern Thailand [12, 13, 14]. Median age of the patients in this study was comparable with the previous report in the Northeastern Thailand, but was younger than CCA patients in western countries [13, 14]. In our cohort, similar proportions of men and women were observed, in contrast with the data reported in the literature where the CCA is predominant in men than women [4, 13, 14, 15]. Variation in primary location, age at the onset and gender predisposition probably reflect differences in risk factors, geography, and potential genetic vulnerability.

Our results confirmed that curative surgery is an important independent prognostic factor in CCA patients. Unfortunately, the majority of CCA patients in our cohort presented at advanced stage. The proportion of metastatic disease at the time of diagnosis was comparable with the previous report in the Northeastern Thailand, however, the rate of curative surgery in this study was higher (15.1% vs. 6.1%) [13]. Although, the percentage of patients underwent curative surgery was still significantly lower than western countries [1, 14]. The difference in surgical management probably resulted from the variation in primary tumor locations, initial tumor staging, surgical expertise, including available heath care resources. However, our study demonstrated that patients with resected localized tumor had extremely better prognosis compared with patients with unresectable or metastatic disease, justifying the importance of improving awareness and screening policies for early detection of this tumor type.

Among the risk factors potentially associated with CCA that were reported, none of our patients had known history of PSC or IBD. There was no evidence of liver fluke infection in our CCA cohort, neither by laboratory examination nor surgical specimen. Moreover, the incidence of cholelithiasis was very low compared with the western studies [14]. However, our study reported a greater proportion of individuals with underlying chronic/post viral hepatitis (over 40%) than previous reported [4, 13]. The reported prevalence of chronic viral hepatitis B in CCA patients varies from 0.2% to 48.6% [16], depending on the epidemiology, study designs, and the method used to identify subjects with chronic hepatitis. In Thailand nationwide study, chronic viral hepatitis were affecting CCA patients in the Southern Thailand for only 1.8% [4]. These discrepancies might be explained by different sources and data collection methods, nonetheless, the prevalence of current (HBsAg+) and past HBV infection (antiHBc+) in CCA patients were much higher than that observed in general population in Southern Thailand at the similar timeframe [17]. Compared to the western countries, as expected, our CCA cohort had a significant greater proportion of chronic/post HBV infection. The remarkably high prevalence of chronic HBV in CCA patients noted in the present study suggests that chronic HBV infection is likely to be one of the important causative factors of CCA development in our region. Many meta‐analysis studies have confirmed the association between HBV and CCA [10, 18, 19]. In another meta‐analysis of 18 case–control studies, pooled risk estimates showed a significant increased risk for iCCA in HBV positive patients (OR = 4.57, 95% CI, 3.43–6.09); whereas a weaker association was reported for eCCA (OR = 2.11, 95% CI, 1.64–2.73) [20]. Similar to our results, the prevalence of antiHBc+ was higher in iCCA, and pCCA, rather than dCCA patients (43.1%, 50%, and 28.6%, respectively, *p* = 0.542).

On the contrary, the proportion of CCA patients with underlying cirrhosis in this study was lower than the proportion in the western studies. Therefore, the increased risk of CCA among HBV patients likely not depends on the development of cirrhosis, but on a direct carcinogenic effect on susceptible cells by these viruses [21].

Furthermore, our study reported a high percentage of individuals with underlying hypertension (36.3%), hyperlipidemia (22.9%), and type 2 diabetes mellitus (17%). The meta‐analysis of case–control studies reported an association between CCA and hypertension (OR = 1.10, 95% CI 0.89–1.37 for iCCA, and OR = 1.21, 95% CI 0.77–1.90 for eCCA) and type 2 diabetes (OR = 1.73, 95% CI 1.47–2.04 for iCCA, and OR = 1.50, 95% CI 1.31–1.71 for eCCA) [20]. Moreover, a positive association between metabolic dysfunction‐associated steatotic liver disease (MASLD) (or a former nomenclature, nonalcoholic fatty liver disease: NAFLD) and CCA had been confirmed by meta‐analysis studies [20, 22]. In spite of the high rate of underlying hyperlipidemia, we could not conclude that the patients had steatotic liver disease based on retrospective data. But taking into account that the significant number of patients with underlying hypertension, hyperlipidemia, and/or diabetes, these risk factors are likely one of the responsible grounds for CCA development in our region.

Many studies suggest a positive association between CCA and cigarette smoking and also alcohol consumption [23]. However, our CCA patients had a high percentage of non‐smokers (57.2%) and non‐alcoholic drinkers (63.9%). Thus, the causal role of smoking and alcohol drinking in the increasing the risk of CCA in Southern Thailand remain unclear, but further cohort studies are required to confirm a causal relationship.

Taken together, our findings suggest that chronic HBV infection and underlying metabolic diseases potentially contribute to the risk of developing CCA in Southern Thailand.

Only 2.2% of all CCA patients were asymptomatic and diagnosed as an incidentally discovered abnormal lesion, detected by screening imaging or blood test. All of them were classified as iCCA, accounting for 4.5% of iCCA patients. This proportion was only one‐fifth of previous reported in the western countries [1]. Therefore, our iCCA cohort had a larger median tumor size, and consequently, lower rate of curative surgical resection and shorter OS [1, 14].

Our findings align with several previous studies demonstrating survival differences based on tumor stage but not primary tumor location. In comparison with eCCA, iCCA groups had a significantly greater tumor size, and higher percentage of metastasis at the time of diagnosis, which can be explained by the location of tumors that are usually asymptomatic during early stage of disease. Conversely, jaundice and cholangitis were predominantly seen in eCCA patients in association with the abnormal liver tests and the elevated CA 19–9, which reflect the nature of tumors that cause biliary tract obstruction. Therefore, the patients with eCCA had a higher proportion of early stage at diagnosis compared with iCCA patients. However, there was no statistical difference in OS between each primary tumor location. One of the main reasons is that most of the patients with eCCA require some additional intervention, that is, biliary drainage, before starting specific treatment for their underlying malignancy. Consistent with our result, systemic chemotherapy was significant independent predictors for reducing mortality rate in unresectable CCA patients (HR 0.30, 95% CI 0.18–0.52 for iCCA and 0.19, 95% CI 0.09–0.38 for eCCA). And palliative biliary drainage was an independent factor in unresectable eCCA patients (HR 0.31, 95% CI 0.15–0.63).

The main strength of the present study is that this is the report from high‐volume tertiary academic hospitals in Southern Thailand to shed the light on our population. Secondly, our CCA patients had a high rate of histopathological confirmation. However, this study has several limitations. Mainly the retrospective nature of the data caused some missing information, especially on the lack of confirmatory diagnostic testing for OV infection. Specifically, urinary antigen detection and serological testing were not performed. The diagnosis of OV infection in our study was based on the absence of OV infection indicators in endoscopic findings, pathological specimens, and stool microscopy. An absence of control group to confirm the strength of the relationship of CCA and predisposing factors. And there was a lack of data regarding genomic phenotypes. Prognostic differences might be explained by tumor genomic differences, for example, IDH1/2, FGFR fusions, BRAF, KRAS, TP53, SMAD4 [1].

## 5 Conclusions

CCA patients were usually given a diagnosis at advanced stage, and their overall survival rate was limited. We observed a significantly greater prevalence of chronic viral hepatitis B infection in CCA patients than previously reported. The results of this study contribute to a better understanding of characteristics of CCA patients in Southern Thailand and to the development of better therapeutic strategies for the future. In addition, an appropriate screening method for bile duct cancer should be established in patients at risk.

## Author Contributions

**Chirawadee Sathitruangsak:** conceptualization (equal), data curation (equal), investigation (lead), resources (lead), writing – original draft (lead), writing – review and editing (equal). **Tanawat Pattarapuntakul:** conceptualization (equal), data curation (equal), investigation (equal), resources (equal), writing ‐ review and editing (equal). **Apichat Kaewdech:** conceptualization (equal), data curation (equal), writing ‐ review and editing (equal). **Tortrakoon Thongkan:** conceptualization (equal), data curation (equal). **Apinya Prisutkul:** investigation (equal), resources (equal). **Phatcharaporn Thongwatchara:** data curation (equal), resources (equal). **Hutcha Sriplung:** conceptualization (equal), project administration (equal), supervision (equal), writing ‐ review and editing (equal). **Chanon Kongkamol:** conceptualization (equal), resources (equal), software (equal). **Kanyanatt Kanokwiroon:** conceptualization (equal), writing ‐ review and editing (equal). **Sumalee Obchoei:** conceptualization (equal), writing ‐ review and editing (equal). **Pimsiri Sripongpun:** conceptualization (lead), data curation (equal), formal analysis (lead), funding acquisition (lead), writing – review and editing (lead).

## Ethics Statement

The study was approved by the Human Research Ethic Committee (HREC) (REC 64‐432‐14‐1) of the Faculty of Medicine, Prince of Songkla University, in accordance with the principle of the Declaration of Helsinki and The International Conference on Harmonization in Good Clinical Practice. This study was granted a waiver of consent due to its retrospective nature, the waiver of consent was also authorized by the HREC.

## Conflicts of Interest

The authors declare no conflicts of interest related to the current study.

## Supporting information


**Figure S1.** Kaplan–Meier Survival Curve for iCCA Patients According to iCCA subtype.

**Table S1.** Characteristics of iCCA Patients According to iCCA Subype.

## Data Availability

All data collected or analyzed during this study are included in this article and its Suppoting Information. Further inquiries can be directed at the corresponding author.
