# Supplementary figures and images for: Impact of Clinical Characteristics and Treatment on Cholangiocarcinoma Prognosis in Southern Thailand
Source: Cancer Med. 2024 Dec 18;13(24):e70491. doi: 10.1002/cam4.70491 (PMC11653158; doi:10.1002/cam4.70491)

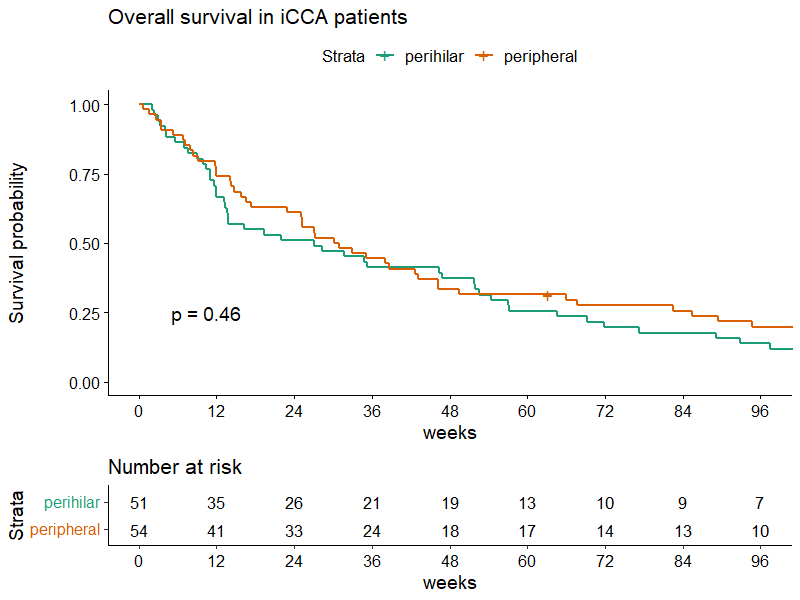

Supplement: Supplementary file 1 — Figure S1. Kaplan–Meier Survival Curve for iCCA Patients According to iCCA subtype. [file CAM4-13-e70491-s001.tiff]
