# Supplementary material for: Impact of Clinical Characteristics and Treatment on Cholangiocarcinoma Prognosis in Southern Thailand
Source: Cancer Med. 2024 Dec 18;13(24):e70491. doi: 10.1002/cam4.70491 (PMC11653158; doi:10.1002/cam4.70491)
Supplement: Supplementary file 2 — Table S1. Characteristics of iCCA Patients According to iCCA Subype. [file CAM4-13-e70491-s002.docx]

|  | **Perihilar iCCA**  **(n = 51)** | **Peripheral iCCA**  **(n = 55)** | **p** |
| --- | --- | --- | --- |
| **Presenting symptoms** | | |  |
| Jaundice | 19 (37.3) | 8 (14.8) | 0.016 |
| Cholangitis | 18 (35.3) | 7 (14.6) | 0.032 |
| Abdominal Pain | 42 (82.4) | 39 (72.2) | 0.316 |
| Anorexia | 25 (49) | 23 (42.6) | 0.642 |
| Pruritus | 7 (13.7) | 1 (1.9) | 0.028 |
| Fever | 12 (23.5) | 4 (7.4) | 0.043 |
| Palpable mas | 4 (7.8) | 7 (13) | 0.591 |
| Nausea/vomiting | 5 (9.8) | 7 (13) | 0.84 |
| **Staging at diagnosis** | | |  |
| Staging (AJCC 8^th^ edition)   - Stage I - Stage II - Stage III - Stage IV | 5 (9.8)  7 (13.7)  9 (17.6)  30 (58.8) | 6 (11.1)  3 (5.6)  3 (5.6)  42 (77.8) | 0.085 |
| Size of primary tumor (median (IQR), cm) | 7.7 (4.8,9.9) | 8.9 (6,10.3) | 0.156 |
| **Baseline laboratory results** |  |  |  |
| Serum tumor marker (median, IQR)   - CA 19-9 - CEA - AFP | 167.9 (17.9,3019.2)  3.4 (2.1,58.8)  3 (2.5,6.3) | 47.6 (14.6,807.9)  5.4 (1.9,32.3)  3.2 (2.3,16.3) | 0.29  0.91  0.879 |
| Total bilirubin (median, IQR) | 1 (0.5,15.3) | 0.6 (0.4,1.4) | 0.007 |
| AST (median, IQR) | 66 (41.2,115.2) | 46.5 (28.2,77) | 0.013 |
| ALT (median, IQR) | 46.5 (25.5,75.2) | 25 (15.2,50) | 0.006 |
| ALP (median, IQR) | 339.5 (193.8,584.2) | 167.5 (122.2,295.5) | < 0.001 |

AFP, alpha-fetoprotein; ALP, alkaline phosphatase; ALT, alanine aminotransferase; AST, aspartate transaminase; CA 19-9, carbohydrate antigen 19-9; CEA, carcinoembryonic antigen;
